# G-quadruplex recognition activities of *E. Coli* MutS

**DOI:** 10.1186/1471-2199-13-23

**Published:** 2012-07-02

**Authors:** Edward A Ehrat, Bradley R Johnson, Jonathan D Williams, Glen M Borchert, Erik D Larson

**Affiliations:** 1 School of Biological Sciences, Illinois State University, Normal, IL, 61790-4120, USA

**Keywords:** DNA repair, G4, Quadruplex DNA, Mismatch repair, MutS

## Abstract

**Background:**

Guanine quadruplex (G4 DNA) is a four-stranded structure that contributes to genome instability and site-specific recombination. G4 DNA folds from sequences containing tandemly repetitive guanines, sequence motifs that are found throughout prokaryote and eukaryote genomes. While some cellular activities have been identified with binding or processing G4 DNA, the factors and pathways governing G4 DNA metabolism are largely undefined. Highly conserved mismatch repair factors have emerged as potential G4-responding complexes because, in addition to initiating heteroduplex correction, the human homologs bind non-B form DNA with high affinity. Moreover, the MutS homologs across species have the capacity to recognize a diverse range of DNA pairing variations and damage, suggesting a conserved ability to bind non-B form DNA.

**Results:**

Here, we asked if *E. coli* MutS and a heteroduplex recognition mutant, MutS F36A, were capable of recognizing and responding to G4 DNA structures. We find by mobility shift assay that *E. coli* MutS binds to G4 DNA with high affinity better than binding to G-T heteroduplexes. In the same assay, MutS F36A failed to recognize G-T mismatched oligonucleotides, as expected, but retained an ability to bind to G4 DNA. Association with G4 DNA by MutS is not likely to activate the mismatch repair pathway because nucleotide binding did not promote release of MutS or MutS F36A from G4 DNA as it does for heteroduplexes. G4 recognition activities occur under physiological conditions, and we find that M13 phage harboring G4-capable DNA poorly infected a MutS deficient strain of *E. coli* compared to M13mp18, suggesting functional roles for mismatch repair factors in the cellular response to unstable genomic elements.

**Conclusions:**

Taken together, our findings demonstrate that *E. coli* MutS has a binding activity specific for non-B form G4 DNA, but such binding appears independent of canonical heteroduplex repair activation.

## Background

Several studies have now connected members of the mismatch repair pathway with the binding and metabolism of non B-form DNA, but in a way that is different than simple heteroduplex repair. Classically defined as a post-replication repair pathway, mismatch repair is initiated through lesion recognition by the MutS homologs. This is accompanied by an ADP-ATP nucleotide exchange that modulates MutS to signal downstream repair (reviewed by [1,2]). Both *E. coli* MutS and the eukaryotic MutS homologs, MSH2/MSH6 (MutSα), have the extraordinary capacity to recognize and initiate repair of unrelated types of DNA damage, such as UV photoproducts [3], 8-oxoguanine pairings [4,5], methylated or platinated DNA [6], and deoxyuracil [7]. In addition to base modifications, DNA structural alterations are recognized. Human MSH2/MSH3 (MutSβ) binds disease-associated hairpins folded from CAG repeats [8], MutSα and MSH4/MSH5 recognize four-stranded Holliday junctions [9,10], and human MutSα binds specifically to G quadruplexes (G4 DNA) found in the immunoglobulin switch regions [9]. This incredibly broad substrate range for the MutS homologs suggests that non-B form DNA structures may be conserved substrates for the complex across species.

**Figure 1:**
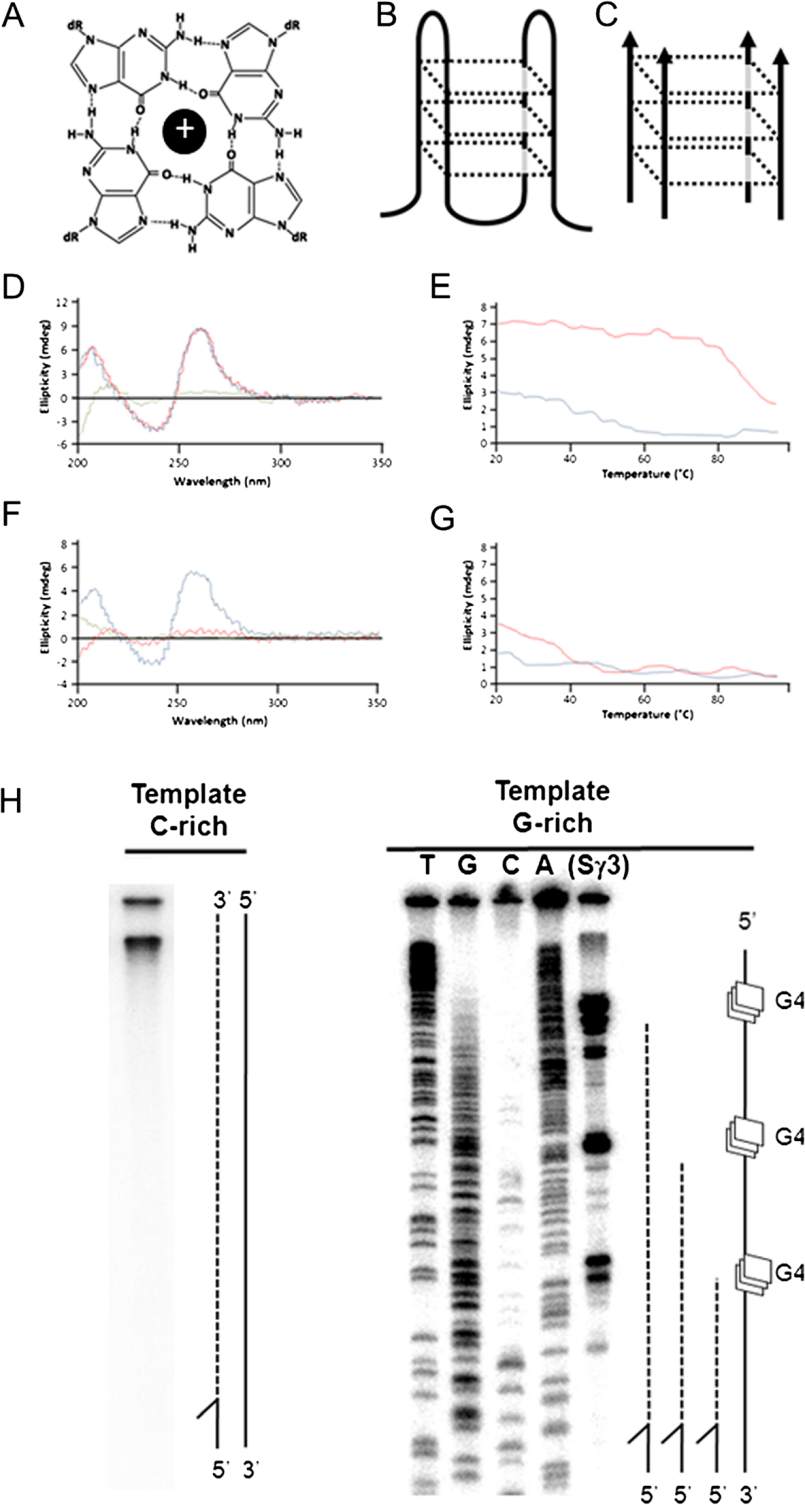
**Formation of G4 DNA structures.** (**A**) Illustration of guanine quartet with each guanine engaged in four hydrogen bonds and a central metallic cation. (**B**) Structural illustration depicting a unimolecular antiparallel G4 DNA. (**C**) Structural illustration depicting an intermolecular parallel G4 formed from guanine rich strands of DNA. (**D**) CD spectrum of TP DNA 49-mer in 50 mM KCl (blue line 25°C, red line 37°C, green line 95°C). (**E**) Melting curve of TP DNA 49-mer molar ellipticity at 263 nm in 50 mM KCl (red line) or 50 mM LiCl (blue line). (**F,G**) As in D,E but TP DNA 49-mer was not pre-folded into G4. (**H**) Primer extension reactions using Klenow fragment and single stranded phagemid template corresponding to the C-rich strand of Sγ3 from pCR2.1-C (left) or the G-rich strand of Sγ3 from pCR2.1-G (right) resolved by denaturing PAGE. Modeled to the right of each image are polymerase extensions (dotted line) on template (solid line) or with G4 sites (squares). Stall sites for G-rich pCR2.1-G map to guanine repeats, determined by cycle di-deoxy sequencing of the G-rich template strand using the extension reaction primer.

Repetitive DNA sequences promote secondary structure formation, and this is affiliated with site-specific gene rearrangements and genome instability (recently reviewed by [11]). G quadruplex (G4 DNA) is a four-stranded structure that folds from guanine-rich repetitive DNA and can adopt multiple potential conformations (Figure 1A-C). Tandemly repetitive guanines support G4 formation under physiological ionic and pH conditions with four hydrogen bonded guanines forming individual tetrads which are stacked on top of one another to form G4 structures [12] (Figure 1A-C). At a minimum, sequences able to sustain G4 structure formation must contain a motif of at least three guanines repeated four times or more with up to a few non-guanine nucleotides separating the motifs [13,14]. Exceeding this minimal standard are repetitive non-coding sequences found throughout the human genome which show extensive G4 folding potential [15,16]. Genome analyses reveal that G4 DNA structures are abundant genome residents and have likely been retained over evolutionary time to facilitate various molecular activities. For instance, pillin gene rearrangement in *Neisseria* depends on upstream G4 structure formation, and sequence disruption or chemical stabilization of G4-folding capacity at these sites interferes with break formation and recombination [17]. In mammals, immunoglobulin gene class switch recombination requires the transcription of intronic switch regions and this results in the formation of co-transcriptional G4 DNAs (structures that have been visualized by electron microscopy [18] and observed by atomic force microscopy [19]). Roles for G4 DNA in gene regulation are supported by informatic analyses identifying G4 capable sequences at upstream gene regulatory regions in S. cerevisiae [20][21], human [22], and prokaryotic [23] genomes. It has been suggested that structure formation in these regions influences gene expression rates because 5’ transcriptional pausing correlates with G4 folding potential [24].

While retention of G4 DNA sequences in diverse genomes suggests genetic roles, structure formation appears to come at a cost to genome stability. In a few examples, minisatellites in mice and humans with high G4 potential are hypervariable (reviewed by [25]), DNA breakpoints in cancer genomes associated with somatic copy number variations regularly map to G4 sequences (reviewed in [26]), and reciprocal translocations of the *c-MYC* gene and the immunoglobulin switch regions occur at G4 sequences (reviewed in [27,28]).

Evidence has emerged that connects the highly conserved mismatch repair proteins with cellular responses to G4 DNA. Human MSH2/MSH6 (MutSα) has been directly visualized by electron microscopy bound to the G-rich and G4 capable strand of expressed immunoglobulin switch regions, enriched in chromatin IPs of the transcriptionally activated switch regions, and shown to specifically recognize G4 oligonucleotide DNA via mobility shift assays [9]. Also in humans, demonstration that FANCJ helicase specifically unwinds G4 DNA [29], coupled with co-immunoprecipitations finding MutS and MutL homologs associated with members of the Fanconi Anemia pathway [30], suggests similar roles with regard to G4 metabolism. While precise functions in structure-associated genome instability are not clear, it does appear likely that non-B form binding is a shared property of the MutS proteins based on the broad substrate range and apparent recognition of G4 DNA binding by the human homologs. Here, we test the model that *E. coli* MutS recognizes G4 DNA structures. Further, we ask if such binding is affiliated with canonical mismatch repair. With mobility shift assays, we identify G4 DNA as a high-affinity substrate for *E. coli* MutS. Interestingly, we also find that G4 DNA and heteroduplex binding constitute separable MutS functions. Our results argue that the genome instability and recombination associated with G4 DNA may involve repair-independent functions of highly conserved mismatch repair proteins.

## Results

### *E. Coli* MutS specifically recognizes G4 DNA

The observations that human MutS homologs specifically recognize four-stranded Holliday junctions [9,10] and G4 DNA [9] prompted us to ask if alternative DNA structure binding is a property of prokaryotic MutS proteins and the mismatch repair pathway. Sequence motifs characteristic of G4 formation have been documented in various prokaryotes, including *E. coli*[23], but highly repetitive G-rich sequences are rare when compared to the more complex genomic structure characteristic of many multicellular eukaryotes. Therefore, we used two model sequences known for strong G4 folding ability and for utility in G4 molecular assays. These sequences are derived from a recombination hot-spot, the G-rich mammalian switch regions, and have utility in binding assays because they readily adopt well-documented G4 DNA structures [9,31-33]. We folded a four-stranded G4 structure from the TP oligonucleotide (Figure 1C) [13,34], and a second longer sequence (564 bp) that supports more complex fold-back G4 structures. TP-G4 DNA was folded using published conditions [14], and structure formation measured by Circular Dichroism (CD). As expected, CD spectra analysis combined with salt-dependent denaturation profile confirmed the formation of G4-DNA by agreeing with anticipated G4 spectra, showing a peak at ~260 nm and a ~240 nm minima [35] with peaks being thermally stable in the presence of K^+^ ion but not Li^+^ ion (Figure 1D-G). Having confirmed that our oligonucleotide-based structure forms G4 by mobility shift (see below) and CD spectrometry, we used this TP-G4 DNA to assay MutS structure-binding properties in vitro.

In the cell, G4 DNA is expected to be unimolecular, a fold-back structure occurring within genomic DNA that has been transiently freed from complement during transcription or replication (Figure 1H). Therefore in order to further examine mismatch repair responses to DNA with potential to adopt non-B form structures, we also examined a longer molecule previously demonstrated to adopt co-transcriptional G4 [9,18,19,36], a fragment of the human Sγ3 switch region. We cloned a 564 bp G-rich region of Sγ3 into M13 and into pCR2.1 phagmids for G4 detection assays (pCR2.1-G or pCR2.1-C, reflecting G-rich strand or C-rich strand with respect to the F1 origin). In these phage, when single-stranded DNA is replicated for packaging and export, one strand is liberated from perfect complement and thus permitted to adopt alternative DNA structures. Template G4 DNA structures present physical blockades to advancing DNA synthesis, which marks structure formation sites [37]. Therefore, we used Klenow extension reactions to detect structure formation by priming the two single-stranded templates with a ^32^P end-labeled oligonucleotide (Additional file 1) just upstream of the Sγ3 sequence. When the extension reactions were resolved by denaturing PAGE, sites of stalled synthesis were evident for the single-stranded G-rich template but not the C-rich complement (Figure 1H). This stalling is very likely to be due to G4 structure formation because polymerase pause sites mapped to guanine repeats, determined by comparison to di-deoxy sequencing of the G-rich template. Furthermore, we always observed products of complete extension when the C-rich complement was used at a template (Figure 1H). This G-rich Sγ3 fragment was therefore used in this study to assay MutS-dependent responses to structure-capable DNA in two strains of *E. coli.*

### Mobility shift assays identify G4 DNA as a substrate for the MutS homologs

**Figure 2:**
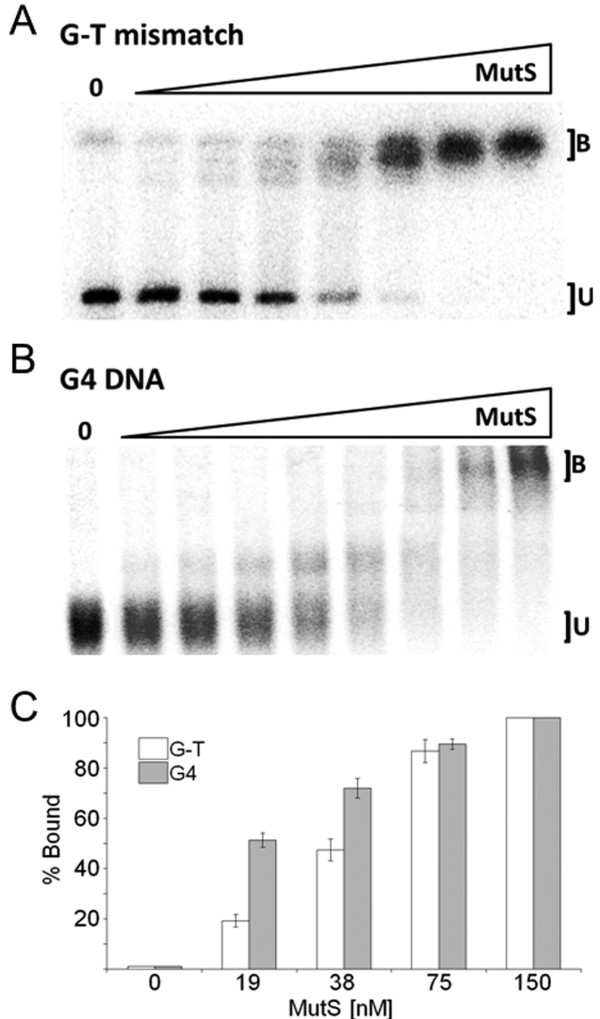
**MutS binds G-T mismatches and G4 DNA.** (**A**) Mobility shift assays demonstrating that wild-type MutS binds G-T mismatches. Lanes contain increasing concentrations of MutS from 4.7 to 300 nM generated by a 1:1 serial dilution. Each lane contains 20 fmols radiolabeled G-T mismatch substrate. B, oligonucleotide bound by MutS; U, unbound. (**B**) As in A except 10 fmols of radiolabeled TP-G4 were utilized as the substrate per reaction with 1.2-150 nM MutS (**C**) MutS binds to G4 DNA with high affinity. Graph depicting the percent of G-T mismatch (white) bound by MutS and percent G4 (grey) bound by MutS. Data (Additional file 2) represent the mean of three independent experiments with standard error.

We first asked if purified *E. coli* MutS was able to recognize G4-containing oligonucleotides and if binding was comparable heteroduplex recognition. Using a mobility shift assay, we observed that MutS specifically recognized an oligonucleotide carrying a single G-T mismatch, a well-known high-affinity substrate for MutS [38,39] (Figure 2A). Identical mobility shift assays containing end-labeled G4 DNA in place of labeled G-T heteroduplex clearly showed a MutS dose-dependent reduction in G4 mobility on native PAGE (Figure 2B). This G4 binding cannot be attributed to non-specific interactions by MutS because all assays contained a 10-fold molar excess of non-specific competitor, an unlabeled homoduplex oligonucleotide (Additional file 1). MutS bound G4 in the low nM range, showing an apparent K_D_ of 18.4 nM, a dissociation constant more than 2-fold lower than what was observed with heteroduplex oligonucleotides under identical assay conditions (Additional file 2). Strikingly, progressively slower migrating bands are observed with increasing MutS concentrations for the G4 but not G-T oligonucleotides (Figure 2B). Multiple MutS dimers may associate with G4 DNA as protein concentration increases, a binding state that is clearly distinct from simple heteroduplex recognition and a binding pattern similarly observed for other G4 binding proteins, such as the RecQ homologs [40]. Regardless, the relative affinity and binding characteristics are remarkably close to human MutSα, which showed in the same assay up to a 4 fold higher affinity for G4 folded oligonucleotide compared to G-T heteroduplex [9]. We conclude from these assays that G4 DNA is a conserved and specific substrate for the MutS homologs, from *E. coli* to humans.

### Mismatch discrimination is not required for G4 recognition

**Figure 3:**
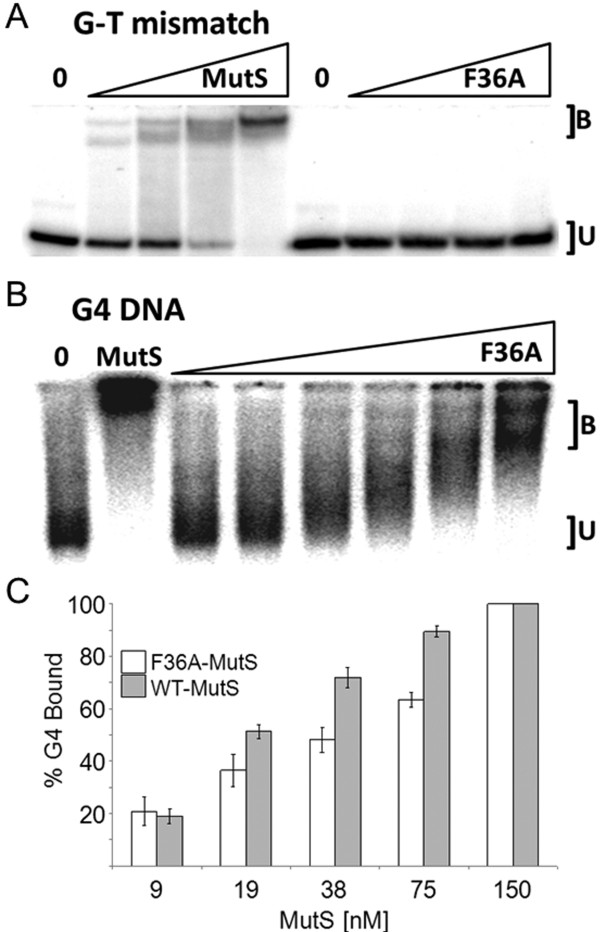
**MutS F36A mutant binds G4 DNA but not G-T mismatches.** (**A**) Mobility shift demonstrating that F36 is required for mismatch binding. From left to right, lanes 1 and 6 contain no MutS protein. Lanes 2–5 and 7–10 contain increasing amounts (19–150 nM) of WT-MutS and F36A-MutS, respectively, obtained by 1:1 serial dilution. Each lane contains 20 fmols radio labeled G-T mismatch substrate. B, oligonucleotide bound by MutS or F36A; U, unbound. (**B**) F36 is dispensable for MutS binding G4. Far left lane “0”, negative control containing no MutS protein; lane 2 “MutS”, positive control containing 75 nM WT-MutS, lanes 3–8 contain increasing amounts of F36A-MutS from 4.7 to 150 nM, obtained by 1:1 serial dilution. Each lane contains 20 fmols of radiolabeled G4. (**C**) Graph comparing WT-MutS and F36A-MutS binding to TP-G4 DNA. White columns, F36A-MutS; grey columns, WT-MutS. Data (Additional file 3) represent the mean of three independent experiments with standard error.

MutS recognition of heteroduplex DNA in vitro and activation of the mismatch repair pathway depends on the highly conserved phenylalanine at position 36. Studies in which this Phe is replaced with Ala (MutS F36A) established this highly conserved residue as critical for heteroduplex recognition and mismatch correction [41]. We therefore reasoned that if G4 structure recognition followed canonical heteroduplex binding activities and repair, MutS binding to G4 DNA should require Phe36. We replaced the Phenylalanine at position 36 of *E. coli* MutS in the pTX412 plasmid (created by Malcolm Winkler [42]) with alanine (MutS F36A) using site-directed mutagenesis. HIS-tagged MutS was over-expressed, and the gene product purified to homogeneity (not shown). Nickel-affinity purified MutS F36A failed to recognize mismatched DNA in mobility shift assays at all protein concentrations tested (Figure 3A), as expected and consistent with the previous study [41]. In contrast, MutS F36A decreased the mobility of G4-containing oligonucleotides in a dose-dependent manner (Figure 3B), demonstrating that the Phe36 residue is not required for G4 DNA recognition. Notably, MutS F36A does not bind G4 DNA as effectively as wild-type MutS. At a concentration of 75 nM, 90% of the labeled G4 is bound by MutS whereas only 63% of the G4 is bound at the same concentration of MutS F36A (Figure 3C, Additional file 3). This attenuated binding may indicate that substitution of Phe36 influences the MutS structure in a way that changes G4 affinity. Nevertheless, it appears likely that Phe36 itself is not critical to the discrimination of G4 DNA structures from properly paired duplex DNA, in sharp contrast to MutS heteroduplex recognition activity.

### MutS remains bound to G4 DNA in the presence of ATPγS

**Figure 4:**
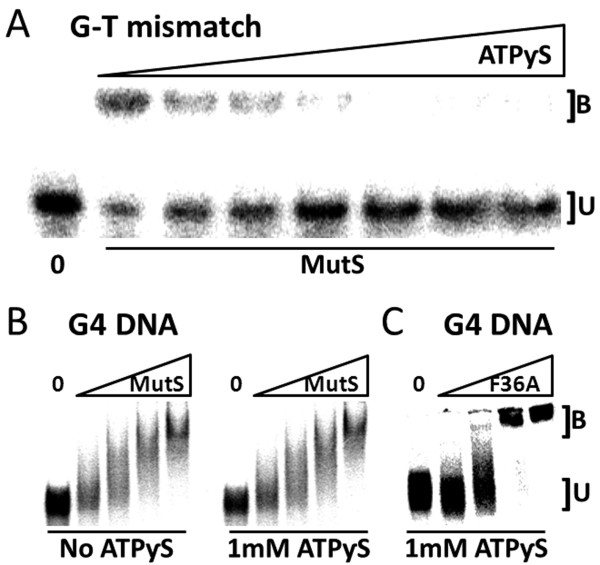
**MutS association with ATP induces the release of G-T mismatches but not G4.** (**A**) MutS dissociates from mismatches in presence of ATPγS. Representative mobility shift assay with radiolabeled G-T mismatch substrate and 60 nM WT-MutS per lane (excluding Lane 1 “0”, no WT-MutS control). Lane 2 contained no ATPγS (positive control) and lanes 3–8 contained increasing concentrations of ATPγS from 31 μM to 1.0 mM generated by a 1:1 serial dilution. B, oligonucleotide bound by MutS; U, unbound. (**B**) MutS binds G4 in presence of ATPγS. Representative mobility shift showing G4 association with WT-MutS with or without ATPγS. Lanes 1 and 6 contain no protein while lanes 2–5 and 7–10 contain increasing amounts of WT-MutS (19, 38, 75, and 150 nM). (**C**) Mobility shift evaluating G4 binding in the presence of 1 mM ATPγS as in B except F36A-MutS is examined in place of WT-MutS. Data (Additional file 4) represent the mean of three independent experiments with standard error.

During mismatch repair, the MutS homologs must release from heteroduplex to activate DNA excision and resynthesis (a conformational change to MutS that is modulated by a separate and distant nucleotide-binding domain). Addition of either ATP or slowly hydrolyzing analogs to binding assays interferes with specific complex formation between MutS and heteroduplexes [43-46] reflecting MutS movement away from the lesion to signal downstream repair. If G4 DNA binding by MutS is accompanied by mismatch repair, addition of ATP to the binding reaction is expected to interfere with G4-MutS complex formation. Using mobility shift assay and sub-physiological concentrations of ATPγS, MutS was not able to shift G-T oligonucleotides, as expected (Figure 4A). ATPγS concentrations above 250 μM resulted in appearance of completely free heteroduplex, and MutS mismatch complex was unstable even at 60 μM ATPγS (Figure 4A). This was not the case for mobility shift assays using G4 oligonucleotide as the substrate. Under identical assay conditions, MutS and MutS F36A binding to G4 DNA was largely unaffected by ATPγS (Figure 4B,C) and K_D_ values for G4 binding were essentially unchanged in the presence and absence of up to 1 mM ATPγS (Additional file 4). While the apparent K_D_ is similar, MutS F36A-G4 binding in the presence or absence of ATPγS does show an altered binding pattern compared to MutS mobility shift of G4 (Figure 2B) which may indicate that the F36A substitution influences the interaction with G4 DNA. Consistent with mobility shift assays in the absence of ATPγS, progressively slower migrating bands are observed with increasing MutS protein (Figures 3,4) indicating that MutS is unresponsive to ATPγS when bound to G4 DNA. Based on a requirement for MutS nucleotide binding status to modulate heteroduplex binding and subsequent repair activities in the mismatch repair pathway, our results support a model whereby MutS is not able to activate ATP-dependent canonical mismatch repair in response to G4 DNA structures.

### MutS F36A facilitates efficient infection by G-rich M13 phage

Mobility shift assays with purified protein and model G4 oligonucleotides in vitro imply that *E. coli* MutS will respond to non-B form DNA structures, independent of repair activation, *in vivo*. While the genome of *E. coli* is mostly coding and generally non-repetitive compared to higher eukaryotes, the genomes of bacteriophage are characteristically non-B form. In particular, filamentous phages, such as M13, infect *E. coli* and replicate by rolling-circle replication using a circular duplex intermediate. By nature of a single-stranded circular genome, these viruses present a DNA molecule that is already non-B form, although we have increased the structure forming potential of M13 even further by introducing the G-rich Sγ3 fragment (demonstrated in Figure 1H to fold into polymerase-stalling structures). We use this M13 variant (called M13-G) and the parent molecule (M13mp18) to ask if the presence of MutS or mismatch repair activities influences phage infection success.

To ask if there are repair-independent and structure-associated roles for MutS in the cell, we infected several *E. coli* strains with M13 phage containing the G4 capable sequence shown in Figure 1H. Importantly, the G-rich strand from the Sγ3 fragment forms robust structures that stall DNA synthesis (Figure 1H), most likely attributed to G4 formation when the molecules are single-stranded circles free from prefect complement. We placed this sequence within M13 (M13-G) such that rolling circle replication will liberate the cloned G4 capable (G-rich) single-strand. We then infected MutS proficient (NM522), deficient NM522 *mutS::TN*10 (JW1), or JW1 expressing MutS F36A (JW1-MutS F36A) to ask if MutS and mismatch repair are needed for infection efficiency when M13 harbors this additional non-viral DNA sequence. Phage infection success for MutS defective strain was measured by counting plaques normalizing to an isogenic MutS proficient strain.

**Figure 5:**
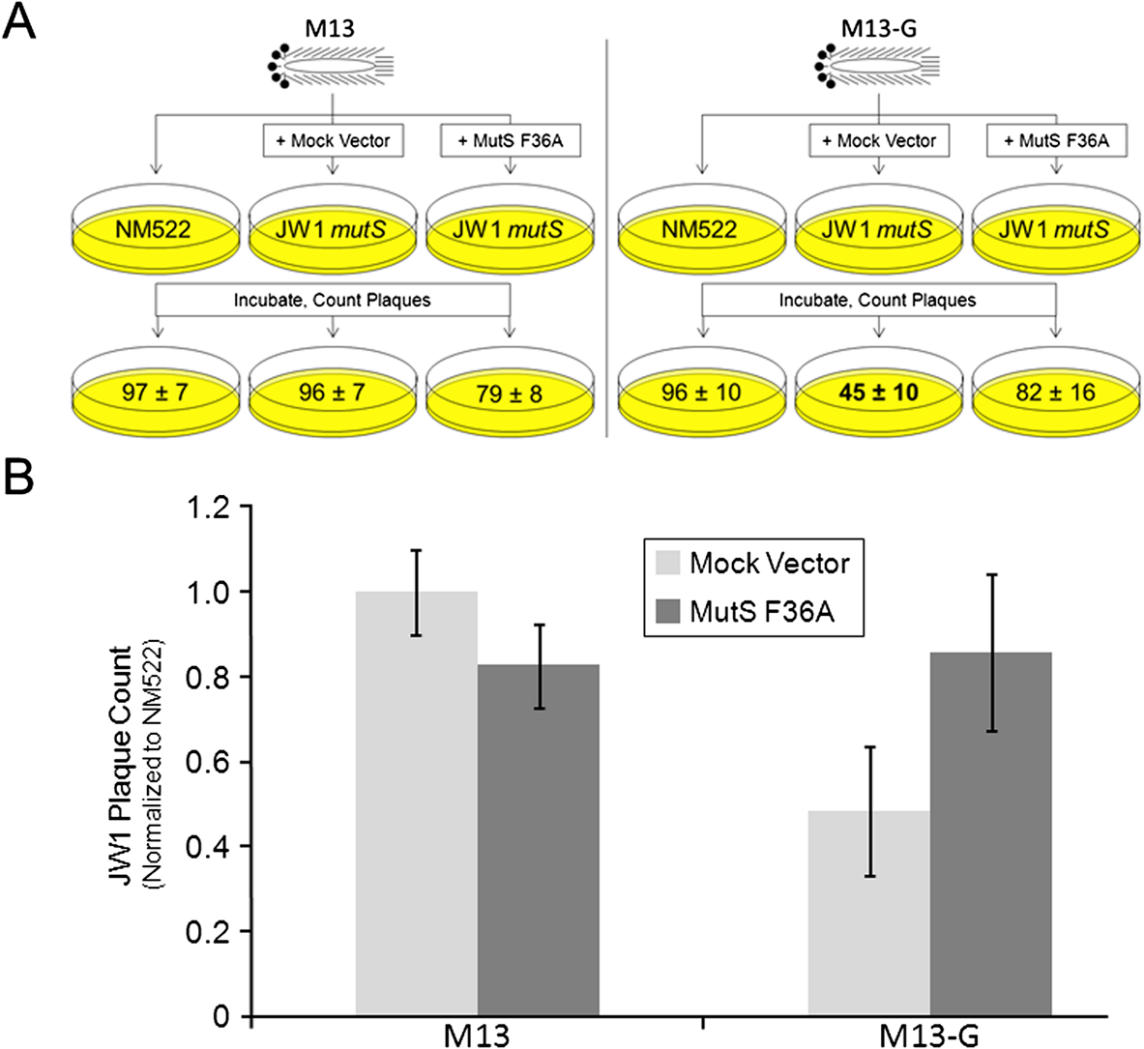
**MutS F36A facilitates efficient infection by G-rich M13 phage.** To ask if MutS G4 binding activity influences phage infection success, we examined the abilities of a M13 variant called M13-G (which contains the G4 capable sequence shown in Figure 1H) and its parent molecule (M13mp18) to infect bacteria in the presence or absence of MutS expression. (**A**) Cartoon depicting plaque assay methodology. MutS proficient (NM522), deficient (NM522 *mutS::TN*10 (JW1)) transformed with empty (Mock Vector), or JW1 expressing MutS F36A from “MutS F36A” vector were infected to ask if MutS influences infection efficiency when M13 harbors a G4 competent sequence. Phage infection success for MutS defective strain was measured by counting plaques/plate. (**B**) Graph depicting results of assay diagrammed in A. Phage infection success for the JW1 MutS defective strain was measured by counting plaques then normalizing to the isogenic MutS proficient strain NM522 (n = 6).

Successful M13 phage infection of bacteria results in the formation of a plaque on LB agar plates. We first titered phage stocks of M13-G and M13mp18 using NM522 and defined the volume required to generate ~100 plaques/plate for each stock. Using identical volumes and conditions, M13mp18 infection showed nearly equal plaques/plate for both NM522 and JW1 (Figure 5) indicating that the MutS protein is not required for efficient infection by M13mp18 phage. In contrast, infection with M13-G resulted in ~50% fewer plaques relative to NM522 infection suggesting that disruption of MutS interferes with phage infection when their genomes harbor exogenous sequences. This is most likely not associated with mismatch repair because expression of MutS F36A in JW1, a mutant defective in mismatch binding and repair but functional for G4 binding ability (Figure 3), resulted in near complete restoration of M13-G phage infection to that of NM522. Although this experiment does not define G4 DNA structures or a G4-specific pathway as the target for MutS in the cell, it does support physiological roles for the MutS complex that are outside of heteroduplex recognition. However, it seems likely that MutS does physically interact with Sγ3 G4 DNA in the cell because immunoprecipitations using MutS antibody weakly enriched for endogenously expressed MutS physically bound to the G-rich Sγ3 expressed in the G4-capable conformation, but not when this sequence placed in the reverse and G4 incapable orientation (Additional file 5). Further, previous EM experiments visualized G4 structure formation of intracellularly expressed murine Sγ3 DNA [18]. Considering that filamentous single-stranded phage DNA is already non-B form, a condition that is enhanced in M13-G due to the additional Sγ3 sequence (Figure 1H), we conclude that MutS has functional roles in the cell associated with DNA structure formation, activities that cannot be easily attributed to mismatch binding or to the canonical mismatch repair pathway.

## Discussion

Our experiments tested the hypothesis that MutS homologs are capable of recognizing non-B form DNA structures, and that such binding is independent of the classically defined mismatch repair pathway. We show here that *E. coli* MutS specifically recognizes G4 DNA with apparent affinity higher to that of G-T mismatched binding. Structural analysis and binding studies of the MutS homodimer previously demonstrated that recognition of mismatched bases is facilitated by one highly conserved Phe at position 36 which facilitates mismatch recognition by stacking with one of the mispaired bases [47,48]. The utility of such a mechanism is a capacity to recognize multiple types of different base mismatches [49]. Substitution of this residue completely blocks mismatch recognition and repair [41]. Nevertheless, MutS F36A retains moderate affinity for G4 structuresas measured by mobility shift assay (Figure 3). Further, expression of a MutS F36A transgene rescued the ability of G4-capable phage to efficiently infect MutS deficient cells (Figure 5). Our findings indicate that the Phe at position 36, required for recognition of heteroduplexes, is not required for structure recognition. This strongly points toward a distinct mechanism for structure recognition by MutS. Even so, it seems most likely that the G4 DNA binding domain of MutS overlaps with that of the heteroduplex domain because G4 binding was reduced by ~20% (Figure 3C) for purified MutS F36A compared to wild type protein. We cannot, however, exclude the possibility that an alternate binding domain is collaterally perturbed by substitution of Phe36 in a way that influences structure binding affinity, or that the MutS F36A protein has lower activity overall.

The licensing of mismatch repair activities by MutS depends upon conformational changes and communication between two distant MutS domains, one for DNA recognition and the other for ATP binding and hydrolysis. In binding experiments nucleotide status modulates MutS complex formation with heteroduplex DNA, and the addition of ATP results in movement of MutS away from the mismatch [50-53]. Consistent with that activity, we did not observe stable MutS-bound G-T mismatched oligonucleotides at ATPγS concentrations tested in mobility shift assays (Figure 4A). In contrast, and under identical reaction conditions, both MutS and MutS F36A remained associated with G4 DNA in the presence of ATPγS (Figure 4B), although the binding pattern appears different for MutS F36A compared to wild-type MutS (compare Figures 2B, 3B and 4B). Therefore it appears that ATP-induced MutS conformational changes that promote heteroduplex release are inadequate to dissociate MutS from G4 DNA. This is a deviation from well-defined binding properties of the MutS homologs, and supports the notion that G4 recognition is not affiliated with mismatch repair as currently defined. Indeed, ATPγS-independent binding to G4 DNA is not confined to *E. coli* MutS as this binding mode is shared with the human MutS homologs. Both Holliday Junctions and G4 DNA are bound with high affinity by human MutSα in the presence of ATP [9]. In contrast with *E. coli* MutS, the human complex may have additional responses to DNA structures because synthetic four-way junctions do not appear to be specific binding substrates (not shown, and [54]). Regardless, it seems likely that the MutS homologs have at least two binding activities; one is affiliated with heteroduplex recognition and ATP-induced mismatch repair, and the other responsive to G4 DNA and independent of methyl-directed mismatch repair.

Genomic regions rich in repetitive guanine are common in higher eukaryotes, but rarer in prokaryotes. G-rich introns located at the 5’ end of expressed loci have been correlated with transcriptional pausing, providing a gene regulation rational for why genetically unstable G-rich DNA may be retained in mammals [24]. However, and relevant to recombination, many hot-spots for genetic rearrangement in mammals contain guanine repeats, such as the immunoglobulin switch region, telomeres, and the rDNA (recently reviewed in [55-57]). This is in striking contrast to prokaryote genomes because, with the exception of the pillin genes in *Neisseria* recombination [17], few recombination-associated sites with strong G4-forming potential have been described. Generally, the prokaryotic loci with G4 potential are short in sequence and located within promoter regions [23]. In other words, G4-capable sequences found within extensive non-coding repetitive elements are rare in prokaryotes, and this is consistent with the more minimalist genomes of single-celled organisms. It is possible that the genetic instability inherent to large sequences with G4 potential is not well tolerated in prokaryotes, and any gene regulatory benefits attributed to G4 structure formation are not sufficient to outweigh the negative consequences associated with the higher potential for genome instability.

Considering the paucity of extensive G4 DNA in prokaryotes, our findings raise an interesting question regarding the reason *E. coli* MutS has such a robust G4 binding ability. We consider it unlikely that the high affinity G4-binding activity of *E. coli* MutS (Figure 2), or even that the mammalian homologs [9,29], has been evolutionarily retained for gene regulation activities through binding promoter-proximal G4 structures. This notion is based exclusively on well-established roles for the complex in repair and recombination. Instead, we find it more plausible that the MutS homologs play yet to be defined roles in alternative DNA structure resolution or site-specific recombination. In humans, MutSα directly interacts with the BLM helicase [58], and BLM has a G4 DNA unwinding activity [32]. Further, human MutSα was shown to inhibit FANCJ unwinding of G4 DNA structures [29] suggesting MutS homologs may play a role in pathway selection for G4 resolution. However, additional studies will be required to determine G4-specific functions in the cell. Nevertheless, the G4 binding we observe with E. coli MutS may reflect a mechanism for discouraging domestication of repetitive DNA elements.

The specific pathways remain to be identified, but it is feasible that MutS helps facilitate replication when structures are present or allows unstable DNA elements to be removed by recombination. Either way, it is clear that the function of MutS in G4 DNA metabolism is not associated with the methyl-directed mismatch repair as currently defined. Mismatch repair factors in E. coli mediated instability at non-B form structure sites within a plasmid-encoded intron from the PKD1 gene [59]. This possibly reflects structure-dependent responses. Such activities may explain the reduced plaques upon M13-G infection of JW1. However, such functions cannot be attributed to mismatch recognition because return of MutS F36A to JW1 recovered M13-G infection success to M13mp18 levels (Figure 5). MutS homologs may contribute to genomic stability at non-B form DNA by rearrangement at DNA structures or another pathway that permits replication through difficult templates. These pathways are not defined for G4 DNA, and further experimentation will be required to determine how MutS proteins participate in cellular responses to non-B form DNA.

## Conclusions

In conclusion, we have found that *E. coli* MutS has a specific G4 structure binding activity. Based on the inability of MutS or MutSα to release from G4 in the presence of ATP, it is likely that non-B form structure recognition follows a pathway distinct from heteroduplex repair. It is interesting to speculate that part of the genome instability associated with G4 DNA may be associated with blockage of canonical repair processes attempting to operate in the vicinity of G4 structures. Additional experiments are required to determine the relationship between conserved G4 binding capacity and mismatch repair activities and the functional consequences to genome stability.

## Methods

### Reagents, oligonucleotides, and strain construction

All oligonucleotides (detailed in Additional file 1) were purchased from Fisher-Operon. End labeling used T4 PNK (New England Biolabs (NEB), Ipswich, MA) and ^32^PγATP (from either MP biomedicals or Perkin Elmer). ATPγS was purchased from MP biomedicals. The *mutS:Tn10* allele from FC1124 (provided by Dr. Pat Foster, Indiana University, Bloomington, IN) was transferred to NM522 (NEB) by P1 transduction to create JW1 then tested for mutator phenotype by nalidixic acid screening. Both PCR and DNA sequencing confirmed MutS interruption and the absence of full length MutS gene in JW1.

### Folding and construction of G4 DNA

Two model G4 DNA substrates were used in this study. Intermolecular G4 was folded from the TP oligonucleotide (Additional file 1), using protocols described previously [9,14,32,34,40]. G4 structure formation was validated by mobility on 6% native PAGE, and by Circular Dichroism (CD) analysis. CD analysis was performed using an Aviv model 215 CD spectrometer with a thermoelectrically controlled cuvette holder. Spectra were taken in 1 cm path quartz cells examining G4 DNA solutions [1 mM] in 10 mM Tris–HCl, pH 7.5, 1 mM EDTA, ±50 mM KCl or LiCl. 200–350 nm UV spectra were recorded at 2 nm increments using a 2 sec averaging time. G4 thermal stability was determined by examining molar ellipticity at 263 nm at increasing temperatures (25–98°C at 2°C increments).

Phagemids containing G4 capable sequences were created using a 564 bp sequence PCR amplified from genomic template (Ramos B cells) with primers specific for the intronic human Sγ3 sequence (Additional file 1). PCR products were gel purified and TOPO cloned (Invitrogen, Carlsbad, CA) into pCR2.1. Orientation of the G-rich or C-rich inserts was verified by DNA sequencing. Purification of single-stranded circular DNA from each phagemid type was accomplished by incubating TOP10 (Invitrogen) transformed cultures with M13K07 helper phage (NEB), followed by single-stranded DNA purification using the manufacturer’s instructions. Polymerase stop assays [37] used Klenow Fragment with manufacturer’s instructions (Fermentas) and single-stranded circular template primed with ^32^P 5’ end-labeled oligonucleotide complementary to a position 5’ of the cloning site in PCR 2.1. Extension reactions were resolved by denaturing PAGE, 7 M Urea 15% 19:1 polyacrylamide, and then imaged by a Storm 840 phosphorimager (Amersham/GE). The identical Sγ3 sequence was transferred from pCR 2.1-G and pCR2.1-C to the multiple cloning site (*Xba*I/*Hin*dIII fragment) of M13mp18 for phage infections assays.

### Protein expression and purification

Over expression and purification of MutS proteins followed previously published protocols using an original MutS expression clone pTX412 created by Malcom Winkler [42] and provided to us by Dr. Peggy Hsieh (NIDDK, Bethesda MD). Substitution mutation of phenylalanine at position 36 for alanine in the MutS gene was performed using the Phusion site-directed mutagenesis kit (Thermo Scientific, Waltham, MA) and 5’ phosphorylated primers (Additional file 1). Phenylalanine at position 36 was replaced with an alanine by changing the codon TTT to GCT. MutS F36A was also subcloned from pET15b into pTrcHis 2B (Invitrogen) for the phage experiments. Sequence verified pTX412 and mutant derivatives were transformed into BL21 Star (DE3) pLysS (Invitrogen) and purifications were performed from over-expressed cultures typically at the 0.5 L scale. At an OD_600_ of 0.5, cells were induced with 1 mM IPTG and allowed to incubate shaking at 37°C for up to 3 hours. Cells were collected by centrifugation, and HIS-tagged proteins were purified by Nickel chromatography (Sigma, St. Louis, MO) as described previously [42]. Purified MutS and MutS F36A was judged to be > 95% pure, as monitored by 6% SDS-PAGE, and the protein quantified by Bradford assay (BioRad, Hercules, CA) using BSA as a standard.

### MutS mobility shift assays

Binding assays used purified MutS and 10-20 fmols of 5’ ^32^P end-labeled G4 or G-T mismatched radiolabeled oligonucleotide in the presence of 200 fmols of homoduplex competitor DNA. G-T heteroduplex and homoduplex DNA were created by annealing HPLC purified oligonucleotides (FisherOperon), which were DNA substrates previously designed by the Hays laboratory for MutS homolog mobility shift assays [60](Additional file 1). Equal mols of each oligo were resuspended in 10 mM TE, 50 mM KCl and incubated in a >90°C water bath that was then allowed to slowly cool to room temperature. Mobility shifts were performed in 20 μl reactions that contained various amounts of MutS protein, 20 mM Tris pH 7.6, 5 mM MgCl_2_, 1 mM DTT, 50 μg/ml BSA, 10 mM KCl and the relevant ^32^P 5’ end labeled substrate. Sucrose was added to 15% and reactions resolved by 6% native PAGE in 0.5X TBE with 5 mM MgCl_2_ and 10 mM KCl. Gels were then transferred to Whatman paper, dried, imaged by phosphorimager Storm 840 (GE Healthcare, Piscataway, NJ) and bands quantified with ImageQuant software (GE Healthcare). All mobility shifts followed the above procedure, with the only exception being the addition of 1 mM ATPγS (MP Biologicals), as indicated. Apparent K_D_ was determined as previously described [9], and graphs of binding data are shown in Additional files 2,3 and 4. The protein concentration where 50% of the labeled substrate is bound was used as the value for apparent K_D_. TP-G4 characteristically shows a more diffuse band compared to heteroduplex oligonucleotide [9,31], and binding was determined by quantifying bands with slower electrophoretic gel mobility, as compared to the no protein control on the same gel. Mobility shifts were repeated with multiple independent protein preparations to verify binding constants, and protein structure and stability for mutant and wild-type MutS protein verified by Circular Dichroism (not shown). MutS and MutS F36A did not bind homoduplex oligonucleotide in mobility shift assays (Additional file 6).

### Phage assays

Identical Ig Sγ3 sequence, described in Figure 1H, was subcloned into M13mp18 (NEB) by digesting pCR2.1-G with *Xba*I and *Hin*dIII restriction fragment, gel purifying the cleavage product, and ligating into M13mp18. Phage cloning was verified by DNA sequencing. All plaque assays and phage purifications followed standard protocols. Larger volume phage stocks of wild-type and M13-G were created by infecting 500 μl of XL2 Blue (Stratagene) at an OD of 0.5 with a single plaque, followed by culturing in 25 ml of LB overnight. Phage were concentrated by standard 2.5 M NaCl/20% PEG precipitation protocol (NEB) and then resuspended in 800 μl of TE buffer. Titers were determined by serial dilution and counting plaques. Appropriate volumes of either M13mp18 or M13-G were added to experimental NM522 to correspond to approximately 100 plaques for each plate. Plaque-forming efficiency for *mutS::Tn*10 NM522 (JW1) for M13mp18 or M13-G is presented relative to plaque-forming efficiency for NM522 (isogenic and MutS proficient). JW1 cells were transformed with MutS F36A under control of the pTrc promoter in pTrcHIS2B or with pTrcHIS2B empty vector, and plaque-forming efficiency determined relative to NM522 infection. In both empty vector and MutS F36A, expression from the pTcr promoter was induced by addition of 1 mM IPTG for 20 minutes prior to phage infection and plated on LB agar containing 1 mM IPTG.

## Abbreviations

Bp, Base pair; IP, Immunoprecipitation; Ig, Immunoglobulin; CD, Circular Dichroism; G4, Guanine quadruplex; MSH, MutS homolog.

## Competing interests

The authors declare that they have no competing interests.

## Authors’ contributions

EAE, experimental design, experimentation, and manuscript preparation. BJ, experimental design and experimentation. JDW, experimental design, and experimentation. GMB, experimental design, experimentation, and manuscript preparation. EDL, research design and manuscript preparation. All authors read and approved the final manuscript.

## Supplementary Material

Additional file 1 Oligonucleotide master list. All oligonucleotides were synthesized commercially (Fisher, Operon). Click here for file

Additional file 2 **MutS binds G4 DNA with high affinity.** Table and graph depicting the percent of G-T mismatch and G4 bound by MutS. Data represent the mean of three independent experiments with standard error. The protein concentration where 50% of the labeled substrate is bound (indicated) was used as the value for apparent KD. Click here for file

Additional file 3 **F36 is not required for MutS binding G4.** Table and graph depicting the percent of G4 bound by MutS and MutS F36A. Data represent the mean of three independent experiments with standard error. The protein concentration where 50% of the labeled substrate is bound (indicated) was used as the value for apparent KD. Click here for file

Additional file 4 **MutS binds G4 in presence of ATPγS.** Table and graph depicting the percent of G4 bound by MutS in the presence or absence of ATPγS. Data represent the mean of three independent experiments with standard error. The protein concentration where 50% of the labeled substrate is bound (indicated) was used as the value for apparent KD. Click here for file

Additional file 5 **MutS immunoprecipitation.** Representative immunoprecipitations performed on E. coli cell lysates. Amplicons were verified by sequencing. Input and IgG controls are indicated. G4 DNA refers to a transformed plasmid containing a portion of the human Sγ3 G4 sequence. Ctl DNA refers to a plasmid identical to the G4 DNA plasmid except the G4 motif is inverted disallowing G4 formation upon IPTG induction. NEB Turbo (Cat# C2986) bacterial cells were transformed with G4 DNA plasmid or control, grown to mid log phase, induced with IPTG, immunoprecipitations performed using anti-MutS or IgG control, and MutS association examined by plasmid-specific PCR. Click here for file

Additional file 6 **Neither MutS nor MutS F36A specifically bind homoduplex DNA.** Mobility shift assay using purified MutS and MutS F36A and labeled homoduplex oligonucleotide. Lane 1, far left, is a negative control with radiolabeled homoduplex DNA but no protein. Lanes 2–4 contain increasing amounts of MutS (38, 75 and 150 nM). Lanes 6–8 contain increasing amounts of MutS F36A (38, 75 and 150 nM). Click here for file
