# Supplementary material for: G-quadruplex recognition activities of E. Coli MutS
Source: BMC Mol Biol. 2012 Jul 2;13:23. doi: 10.1186/1471-2199-13-23 (PMC3437207; doi:10.1186/1471-2199-13-23)
Supplement: Additional file 1 — Oligonucleotide master list. All oligonucleotides were synthesized commercially (Fisher, Operon). [file 1471-2199-13-23-S1.docx]

**Additional file 1 - Table S1: Oligonucleotide master list.** All oligonucleotides were synthesized commercially (Fisher, Operon).

EDL 462 GTAAAACGACGGCCAG M13 Forward Primer for sequencing

EDL 492 AGTGTGATGGATATCTGCAG Forward primer for stop assay

EDL 454 AACAGTGGACCGAGTGACGTTGC Forward primer for amplification of Sγ3 region

EDL 456 AGCTCCAGGAGCTGCCACCTG Reverse primer for amplification of Sγ3 region

TP G4 TGGACCAGACCTAGCAGCTATGGGGGAGCTGGGGAAGGTGGGAATGTGA

*F36A forward (EDL 424) P - CCGGATGGGTGATGCTTATGAACTGTTTTATGAC

*F36A reverse (EDL 425) P – TAAAACAGCAGGATCTCGGGATGCTGGGCTTTC

MUTsFseq ATGAGTGCAATAGAAAATTTCGACGCCC

MUTsRseq TTACACCAGGCTCTTCAAGCGATAAATCC

MUTsFseqInt GGGCTATAACGAAGAGCTGGATGAGTGG

MUTsRseqInt CCACTCATCCAGCTCTTCGTTATAGCCC

MUTs5’seq ATACGCACAACTTTGCGCTCAACC

MUTs3’seq CCCACTATTTCGAGCTGACCCAGTTACC

MUTsMidRseq TAGCTCTGGAATGATGTAGCGCTCGG

G-T mismatch CTAGCAAGCTTTCGATTCTAGAAATTCGGC

Homoduplex CTAGCAAGCTTTCAATTCTAGAAATTCGGC

GT mismatch/Homoduplex complement GCCGAATTTCTAGAATTGAAAGCTTGCTAG

* 5’ phosphorylated primers
