# Supplementary material for: G-quadruplex recognition activities of E. Coli MutS
Source: BMC Mol Biol. 2012 Jul 2;13:23. doi: 10.1186/1471-2199-13-23 (PMC3437207; doi:10.1186/1471-2199-13-23)

**Additional file 2. MutS binds G4 DNA with high affinity.** Table and graph depicting the percent of G-T mismatch and G4 bound by MutS. Data represent the mean of three independent experiments with standard error. The protein concentration where 50% of the labeled substrate is bound (indicated) was used as the value for apparent KD.


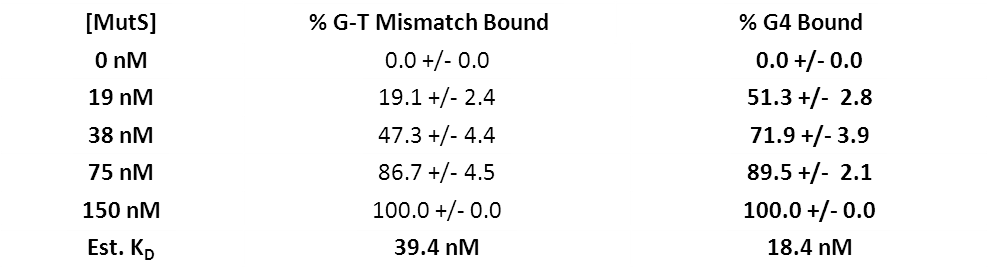


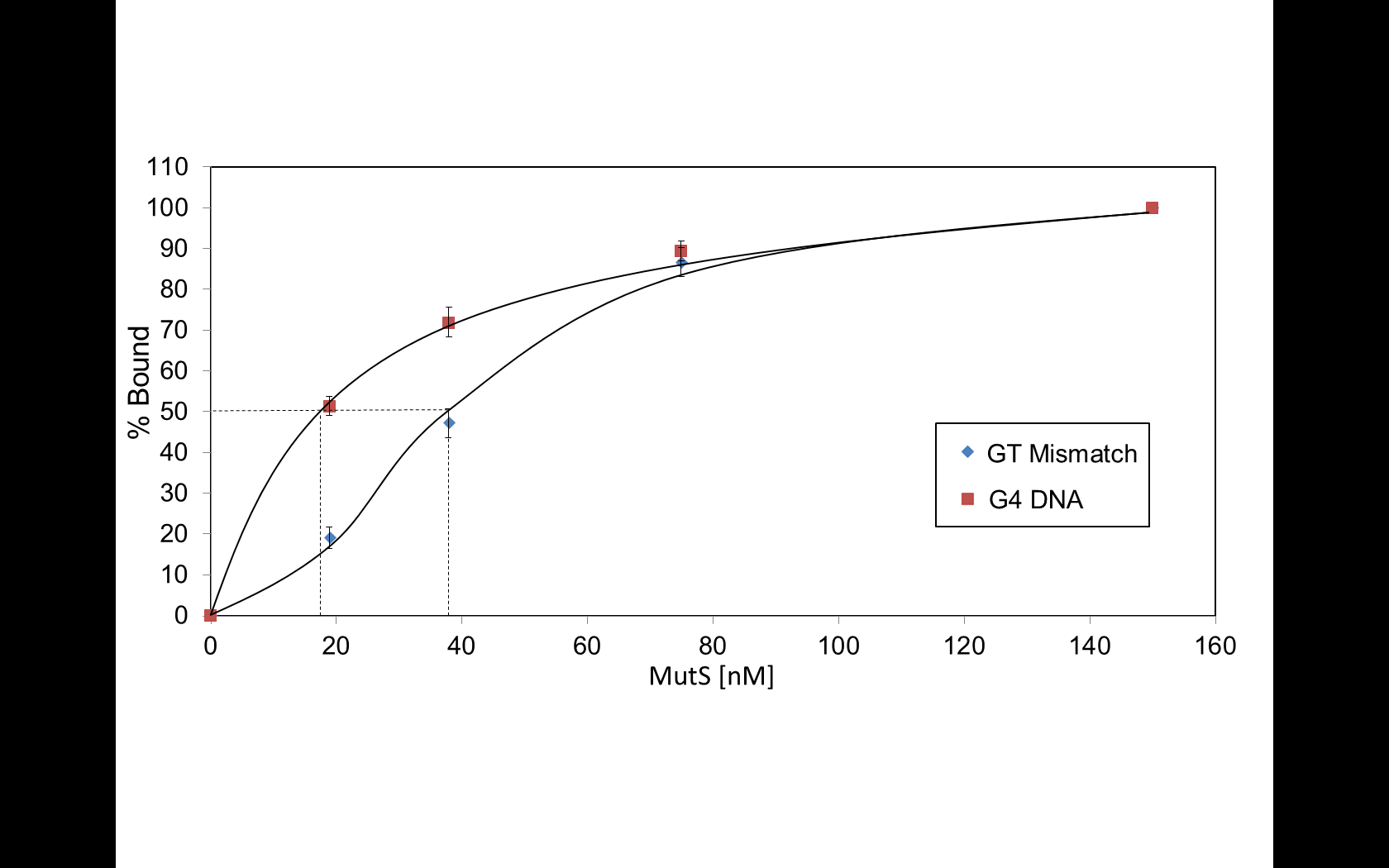

Supplement: Additional file 2 — MutS binds G4 DNA with high affinity. Table and graph depicting the percent of G-T mismatch and G4 bound by MutS. Data represent the mean of three independent experiments with standard error. The protein concentration where 50% of the labeled substrate is bound (indicated) was used as the value for apparent KD. [file 1471-2199-13-23-S2.docx]
