# Supplementary material for: G-quadruplex recognition activities of E. Coli MutS
Source: BMC Mol Biol. 2012 Jul 2;13:23. doi: 10.1186/1471-2199-13-23 (PMC3437207; doi:10.1186/1471-2199-13-23)

**Additional file 3. F36 is not required for MutS binding G4.** Table and graph depicting the percent of G4 bound by MutS and MutS F36A. Data represent the mean of three independent experiments with standard error. The protein concentration where 50% of the labeled substrate is bound (indicated) was used as the value for apparent KD.


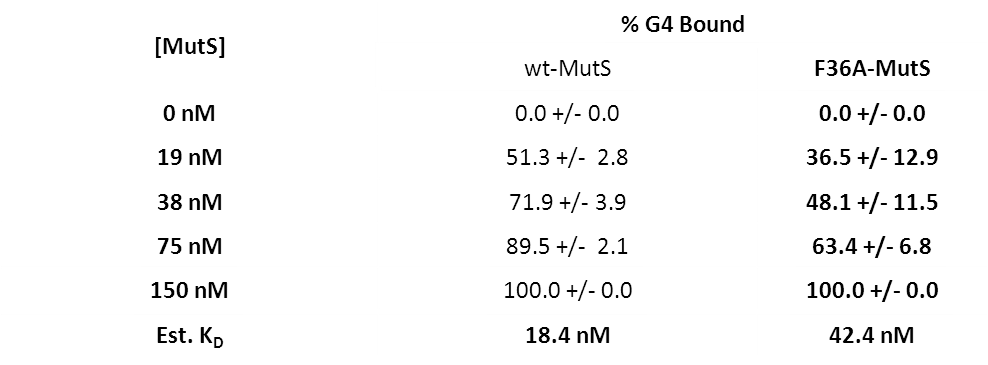


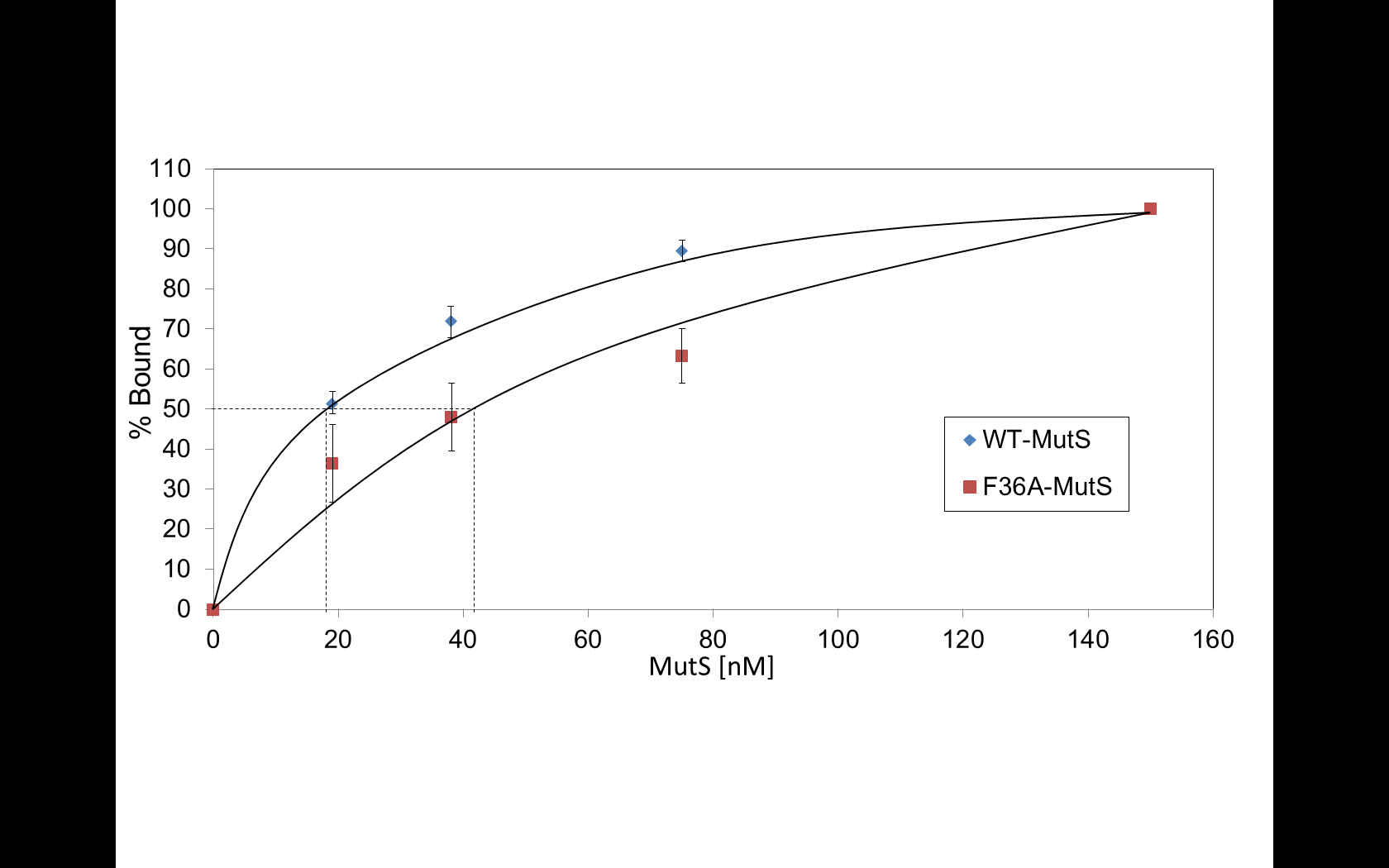

Supplement: Additional file 3 — F36 is not required for MutS binding G4. Table and graph depicting the percent of G4 bound by MutS and MutS F36A. Data represent the mean of three independent experiments with standard error. The protein concentration where 50% of the labeled substrate is bound (indicated) was used as the value for apparent KD. [file 1471-2199-13-23-S3.docx]
