# Supplementary material for: G-quadruplex recognition activities of E. Coli MutS
Source: BMC Mol Biol. 2012 Jul 2;13:23. doi: 10.1186/1471-2199-13-23 (PMC3437207; doi:10.1186/1471-2199-13-23)

**Additional file 4. MutS binds G4 in presence of ATPγS.** Table and graph depicting the percent of G4 bound by MutS in the presence or absence of ATPγS. Data represent the mean of three independent experiments with standard error. The protein concentration where 50% of the labeled substrate is bound (indicated) was used as the value for apparent KD.

| **[MutS]** | **% G4 Bound by wt-MutS** | |
| --- | --- | --- |
|  | **No ATPγS** | **1 mM ATPγS** |
| **0 nM** | **0.0 +/- 0.0** | **0.0 +/- 0.0** |
| **19 nM** | **51.3 +/- 2.8** | **35.7 +/- 4.0** |
| **38 nM** | **71.9 +/- 3.9** | **66.0 +/- 4.8** |
| **75 nM** | **89.5 +/- 2.1** | **87.4 +/- 4.6** |
| **150 nM** | **100.0 +/- 0.0** | **100.0 +/- 0.0** |
| **Est. K_D_** | **18.4 nM** | **27.3 nM** |


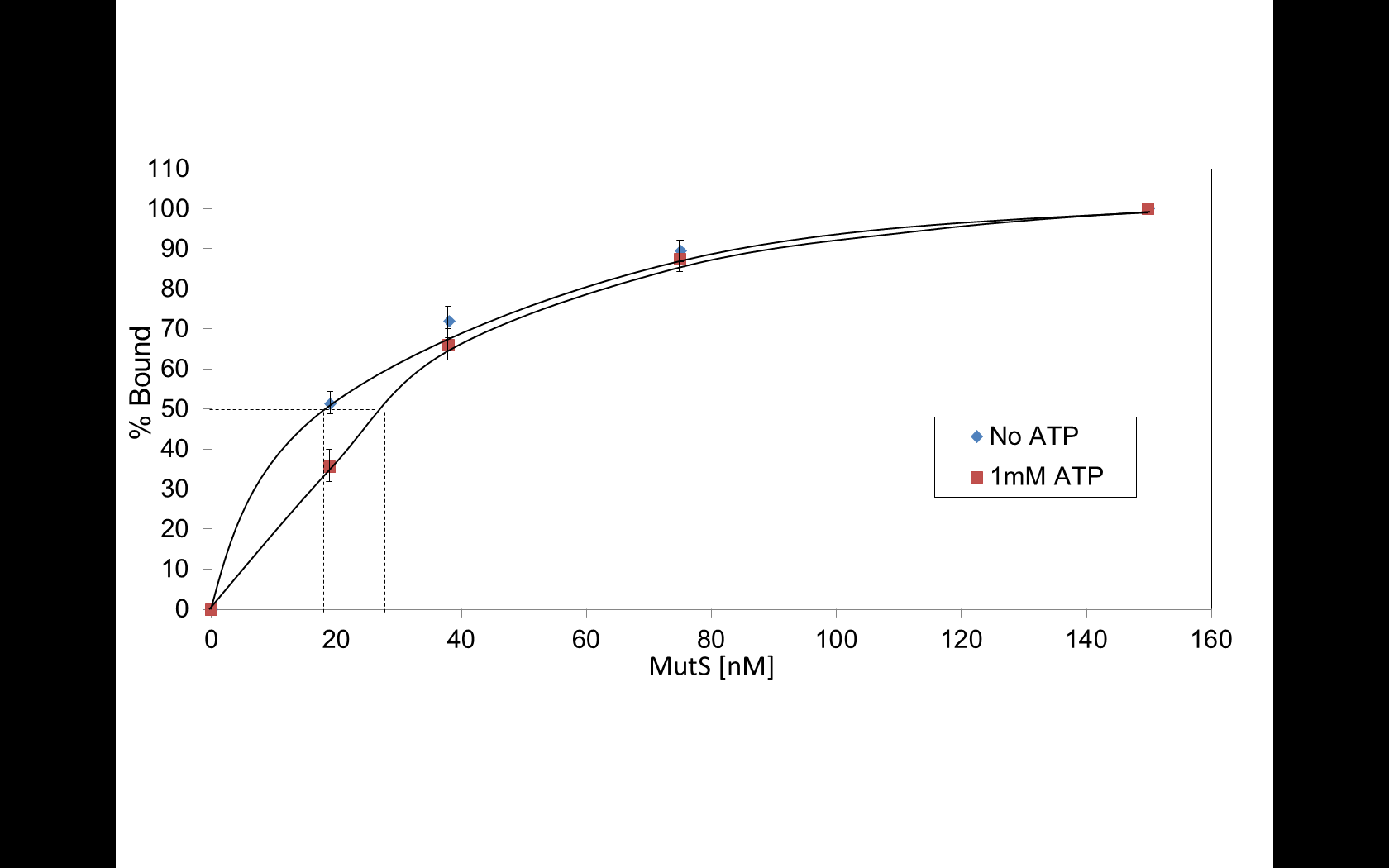

Supplement: Additional file 4 — MutS binds G4 in presence of ATPγS. Table and graph depicting the percent of G4 bound by MutS in the presence or absence of ATPγS. Data represent the mean of three independent experiments with standard error. The protein concentration where 50% of the labeled substrate is bound (indicated) was used as the value for apparent KD. [file 1471-2199-13-23-S4.docx]
