# Supplementary material for: G-quadruplex recognition activities of E. Coli MutS
Source: BMC Mol Biol. 2012 Jul 2;13:23. doi: 10.1186/1471-2199-13-23 (PMC3437207; doi:10.1186/1471-2199-13-23)
Supplement: Additional file 5 — MutS immunoprecipitation. Representative immunoprecipitations performed on E. coli cell lysates. Amplicons were verified by sequencing. Input and IgG controls are indicated. G4 DNA refers to a transformed plasmid containing a portion of the human Sγ3 G4 sequence. Ctl DNA refers to a plasmid identical to the G4 DNA plasmid except the G4 motif is inverted disallowing G4 formation upon IPTG induction. NEB Turbo (Cat# C2986) bacterial cells were transformed with G4 DNA plasmid or control, grown to mid log phase, induced with IPTG, immunoprecipitations performed using anti-MutS or IgG control, and MutS association examined by plasmid-specific PCR. [file 1471-2199-13-23-S5.docx]

**Additional file 5. MutS immunoprecipitation.** Representative immunoprecipitations performed on *E. coli* cell lysates. Amplicons were verified by sequencing. Input and IgG controls are indicated. G4 DNA refers to a transformed plasmid containing a portion of the human Sƴ3 G4 sequence. Ctl DNA refers to a plasmid identical to the G4 DNA plasmid except the G4 motif is inverted disallowing G4 formation upon IPTG induction. NEB turbo (NEB) bacterial cells were transformed with G4 DNA plasmid or control, grown to mid log phase, induced with IPTG, immunoprecipitations performed using anti-MutS or IgG control, and MutS association examined by plasmid-specific PCR.


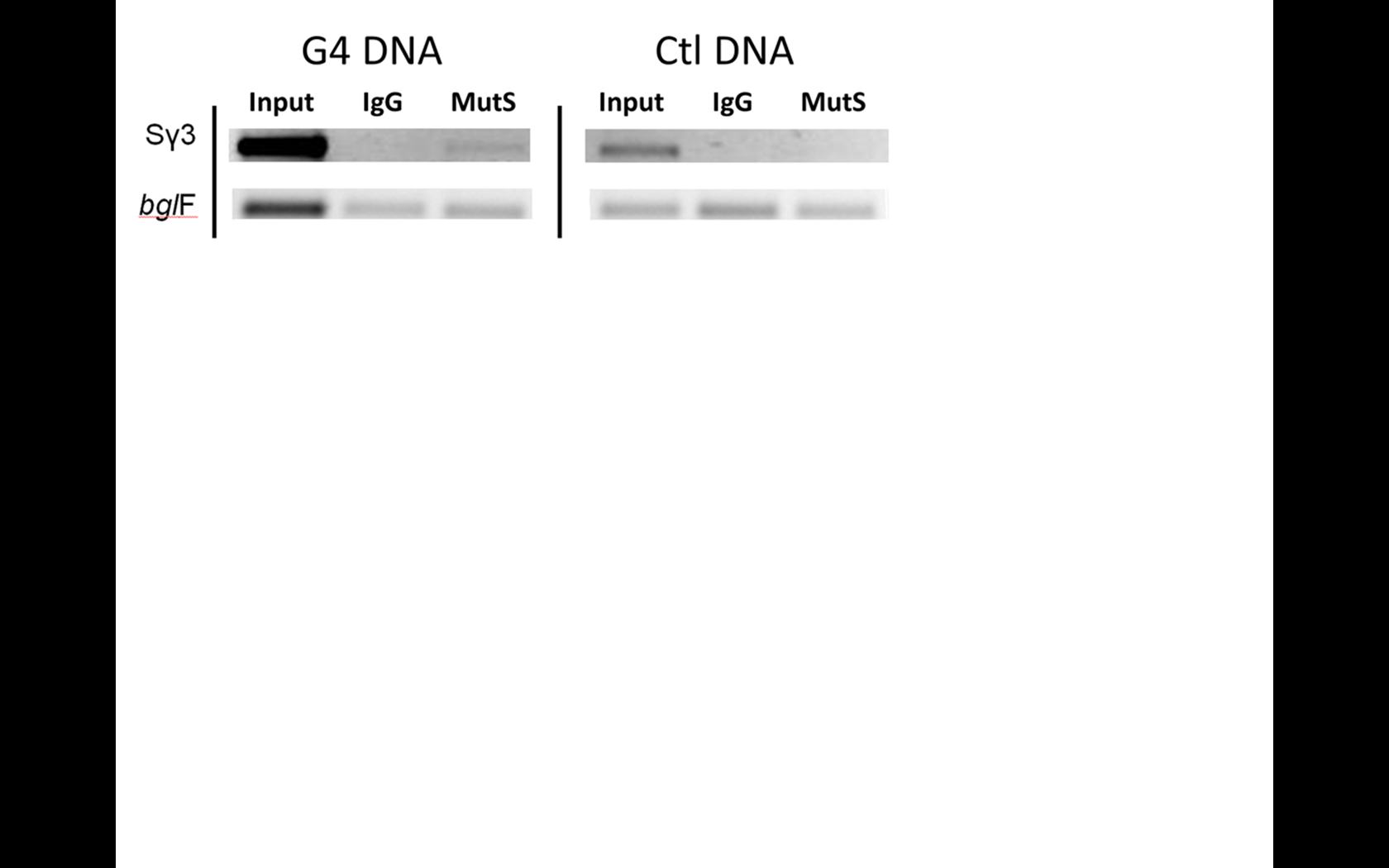


**Additional file 5 methods. MutS Immunoprecipitation.** In all experiments (NEB turbo, WT MutS IP), bacteria carrying plasmids as appropriate were grown to mid exponential phase (optical density A600 0.4 to 0.6) in LB then induced with 1mM IPTG. In vivo cross-linking was initiated by the addition of formaldehyde (final concentration of 1%) to cultures. After 10 min, cross-linking was quenched by addition of glycine (final concentration of 0.5M). Cells were then harvested from 25 ml culture by centrifugation, washed twice with Tris-buffered-saline (pH 7.5), resuspended in 1mL lysis buffer (10mM Tris [pH 8.0], 20% sucrose, 50 mM NaCl, 10 mM EDTA, 10 mg of lysozyme per ml) and incubated at 37°C for 30 min. Following lysis, 4 ml of immunoprecipitation buffer (50 mM HEPES-KOH [pH 7.5], 150mM NaCl, 1mM EDTA, 1% Triton X-100, 0.1% sodium deoxycholate, 0.1% sodium dodecylsulfate [SDS]) and 1X Protease Inhibitor Cocktail (Thermo Scientific) were added. Cellular DNA was then sheared by sonication to an average size of 500 to 1,000 bp. Cell debris was removed by centrifugation and the supernatant was retained for restriction digestion with Pvu II and Bgl I (NEB) at 37°C overnight. IP primary antibodies were normal mouse IgG negative control (Santa Cruz Biotechnology, Santa Cruz, Ca, sc-2025) and anti-MutS (Gene Check, Ft. Collins, CO). 900 µL of Dilution Buffer (10mM Tris [pH 8.0], 150mM NaCl, 10 mM EDTA, 0.1% sodium dodecylsulfate [SDS], 1% Triton X-100 and 1X Protease Inhibitor Cocktail (Thermo Scientific), 40 µL of Santa Cruz Protein A/G Plus Agarose Beads and 6 µL of Invitrogen Herring Sperm DNA (Cat # 15634) were added to individual 100 µl supernatant aliquots, rotated at 4°C for 1 hr, centrifuged at 5000g at 4°C for 2 min, supernatants transferred to new 1.5 mL tubes, 5 µg of each Ab added to the appropriate supernatant tube then rotated over night at 4°C. Next, 40 µL of Santa Cruz Protein A/G Plus Agarose Beads and 6 µL of Invitrogen Herring Sperm DNA (Cat # 15634) were added to individual supernatant aliquots, rotated at 4°C for 1 hr, centrifuged at 5000g at 4°C for 2 min then supernatants removed. Beads were repeatedly centrifuged and washed: 2x with low salt solution (10mM Tris [pH 8.0], 15mM NaCl, 10 mM EDTA, 0.1% sodium dodecylsulfate [SDS], 1% Triton X-100 and 1X Protease Inhibitor Cocktail (Thermo Scientific)), once with LiCl solution (10mM Tris [pH 8.0], 25mM LiCl, 10 mM EDTA, 1% NP40 detergent, 1% Na Deoxycholate and 1X Protease Inhibitor Cocktail (Thermo Scientific)), and 2x with TE buffer. Following this, antibody complexes were eluted from beads by performing two identical elutions with 200 µL of Elution Buffer (1% SDS, 50mM Tris [pH 8.0], 10 mM EDTA), vortexing the samples, rotating them at room temperature for 15 min, centrifuging at full speed for 2 min, and then transfering the supernatants to new tubes. Following this, 16.67 µL of 5M NaCl was added to each 400 µL tube and crosslinks were reversed by incubating samples at 65°C overnight, enriched DNA template was then analyzed by standard PCR and agarose electrophoresis.
