# Supplementary material for: G-quadruplex recognition activities of E. Coli MutS
Source: BMC Mol Biol. 2012 Jul 2;13:23. doi: 10.1186/1471-2199-13-23 (PMC3437207; doi:10.1186/1471-2199-13-23)
Supplement: Additional file 6 — Neither MutS nor MutS F36A specifically bind homoduplex DNA. Mobility shift assay using purified MutS and MutS F36A and labeled homoduplex oligonucleotide. Lane 1, far left, is a negative control with radiolabeled homoduplex DNA but no protein. Lanes 2–4 contain increasing amounts of MutS (38, 75 and 150 nM). Lanes 6–8 contain increasing amounts of MutS F36A (38, 75 and 150 nM). [file 1471-2199-13-23-S6.docx]

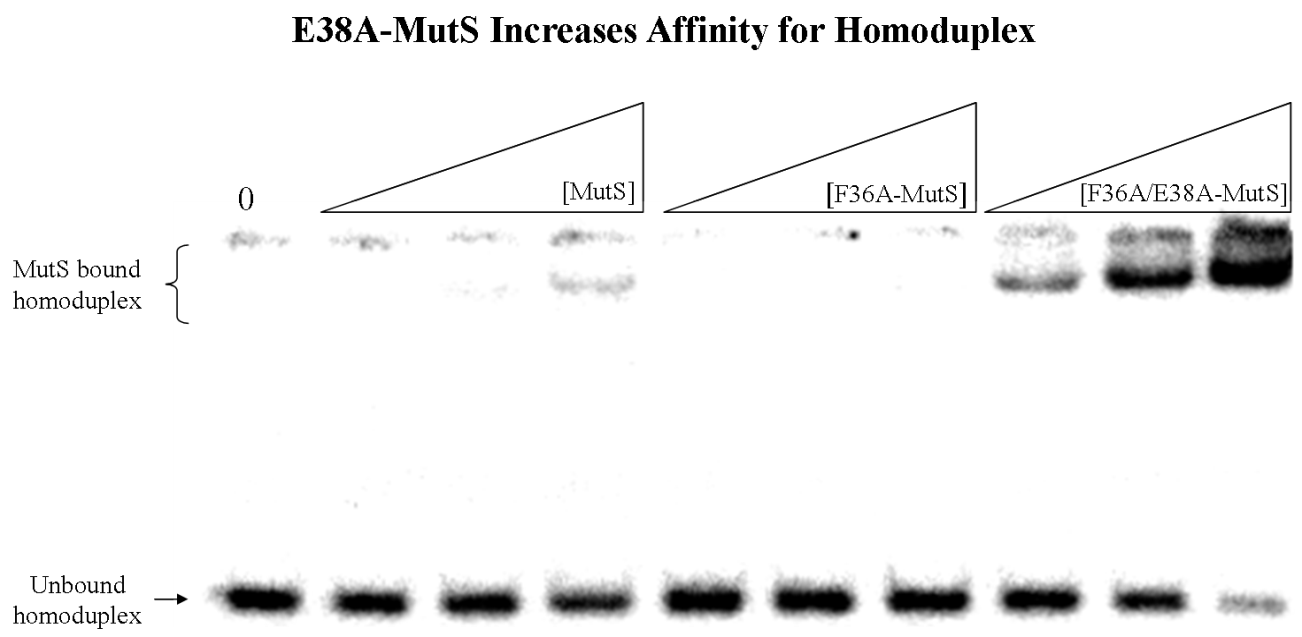


**Additional file 6 - Neither MutS nor MutS F36A specifically bind homoduplex DNA.**

Mobility shift assay using purified MutS and MutS F36A and labeled homoduplex oligonucleotide. Lane 1, far left, is a negative control with radiolabeled homoduplex DNA but no protein. Lanes 2-4 contain increasing amounts of MutS (38, 75 and 150 nM). Lanes 5-7 contain increasing amounts of MutS F36A (38, 75 and150 nM).
